# Multiple tuberculous nodules with metachronous changes: a case report

**DOI:** 10.1186/1756-0500-6-320

**Published:** 2013-08-12

**Authors:** Munehisa Fukusumi, Tatsuya Ibe, Shinjiro Takeoka, Kazushige Wakuda, Atsuto Mouri, Yoichiro Hamamoto, Mitsuhiro Kamimura

**Affiliations:** 1 Department of Pulmonary Medicine, National Hospital Organization Disaster Medical Center, 3256 Midori-cho, Tachikawa, Japan

**Keywords:** Pulmonary tuberculosis, Multifocal nodules, Spontaneous regression

## Abstract

**Background:**

Spontaneous regression of lesions occurs in non-infectious benign diseases, such as sarcoidosis, as well as in infectious diseases, such as tuberculosis. Lung cancer and malignant lymphoma, on the other hand, rarely follow a similar course. We report a rare case of lung tuberculosis that presented with multiple nodules with metachronous changes in size.

**Case presentation:**

We describe the case of a 50-year-old immunocompetent Japanese man with pulmonary tuberculosis in the form of multifocal nodules. He came to our hospital because of a chest X-ray abnormality. During the course of observation, some nodules reduced while others enlarged in size. Two years after the first visit, fever and pleural effusion appeared. The sputum examination turned out to be positive for tuberculosis. A course of anti-tubercular agents resolved the pleural effusion and multifocal nodules.

**Conclusion:**

Differences in the manner of granuloma formation suggest that the local immune response can be different even in the same lung field.

## Background

Spontaneous regression of lesions occurs in non-infectious benign diseases, such as sarcoidosis [1], as well as in infectious diseases, such as tuberculosis. Lung cancer and malignant lymphoma, on the other hand, rarely follow a similar course [2,3]. Local immune response and granuloma formation are the main factors contributing to spontaneous regression of the lesions. Here, we report a Japanese man who suffered from tuberculosis of the lung in the form of multiple nodules that showed metachronous changes in size.

## Case presentation

**Figure 1:**
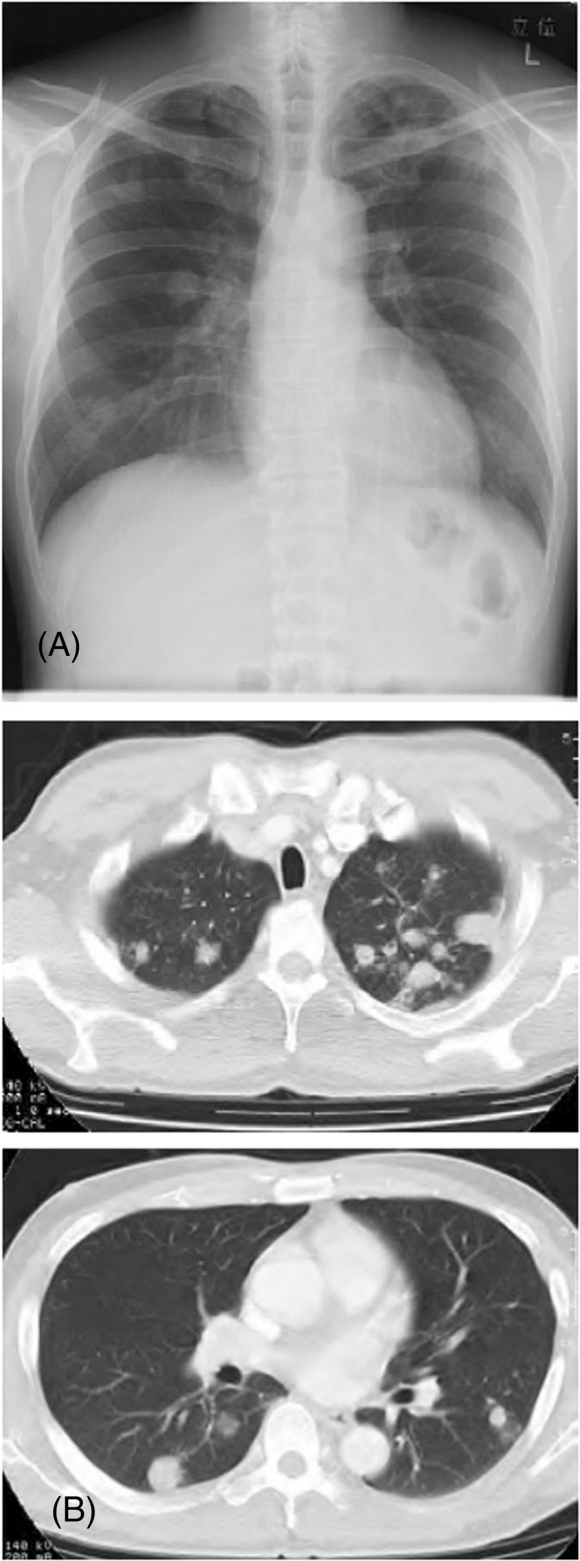
**Initial chest X-ray and CT.** Chest X-ray **(A)** and computed tomography (CT) **(B)** images showing multifocal nodules scattered over the lung fields bilaterally.

A 50-year-old male high school teacher with no significant past medical history presented to our hospital in October 2005 because of an abnormal shadow on chest X-ray. He had no cough, sputum or weight loss and no history of inhalation of dust. He had a history of smoking one pack of cigarettes a day for 30 years. Physically, he was afebrile, with normal breath sounds and no palpable surface lymph nodes. His white blood cell count was 5100/μl with a normal differential count. His one-hour ESR value was 24 mm, and C-reactive protein was 0.13 mg/dl. Liver and kidney function was normal. Carcino-embryonic antigen (CEA) was within the normal range. Angiotensin converting enzyme (ACE) was 12.5 IU/l, and cryptococcal antigen was negative. Stool samples were negative for occult blood. Sputum smear examination was negative for acid-fast bacterium (AFB) and 8-week cultures for mycobacterium were negative (Table 1). The Mantoux purified protein derivative (PPD) test was not performed because he refused frequent visits to the hospital. Chest X-rays and CT scan revealed multifocal nodules scattered over both lung fields. Hilar and mediastinal lymphadenopathy was not found (Figure 1). Bronchoscopic lavage samples were negative for AFB on smear testing, 8-week culture and PCR-TB/MAC, and also negative on cytology and transbronchial lung biopsy (TBLB). Neoplasms, including non-Hodgkin’s lymphoma and lung cancer, were considered as the most probable differential diagnosis. However, these could not be excluded at first, because the patient refused further workup, such as CT-guided or video-assisted thoracoscopic biopsy. Hence, he was followed up by X-rays and CT scans, without treatment. During the course of follow-up, the nodules in both the upper lung fields and the right middle field were all seen to reduce in size. Septic emboli and malignancy were ruled out due to the lack of evidence of inflammation and the spontaneous regression in size. *Paragonimus westermani* was also ruled out due to the absence of a history of eating Japanese mitten crab or crayfish and a low eosinophil count. Antineutrophil cytoplasmic antibody (ANCA)-associated vasculitis was not a possible diagnosis because he did not have any symptoms of sinusitis, glomerulonephritis and mononeuritis multiplex. A benign granulomatous disease, such as nodular sarcoidosis, was thus the most probable diagnosis.

**Table 1:** Laboratory data

Hematology	
WBC	5100 /μl
Neutro	68%
Eosino	2.0%
Mono	5.0%
Lympho	24%
RBC	452 × 10^4^/μl
Hb	14.2 g/dl
Plt	36.4 × 10^4^/μl
Biochemistry	
TP	7.4 g/dl
Alb	4.2 g/dl
AST	15 IU/l
ALT	10 IU/l
LDH	140 IU/l
BUN	14 mg/dl
Cr	0.83 mg/dl
Serology	
ESR	24 mm/1h
CRP	0.13 mg/dl
ACE	12.5 IU/l
CEA	2.1 ng/ml
RF	(−)
ANA	×20
Cryptococcal antigen	(−)
Fecal occult blood	(−)
Sputum	
Mycobacterium	(−)
TB:PCR	(−)
MAC:PCR	(−)

**Figure 2:**
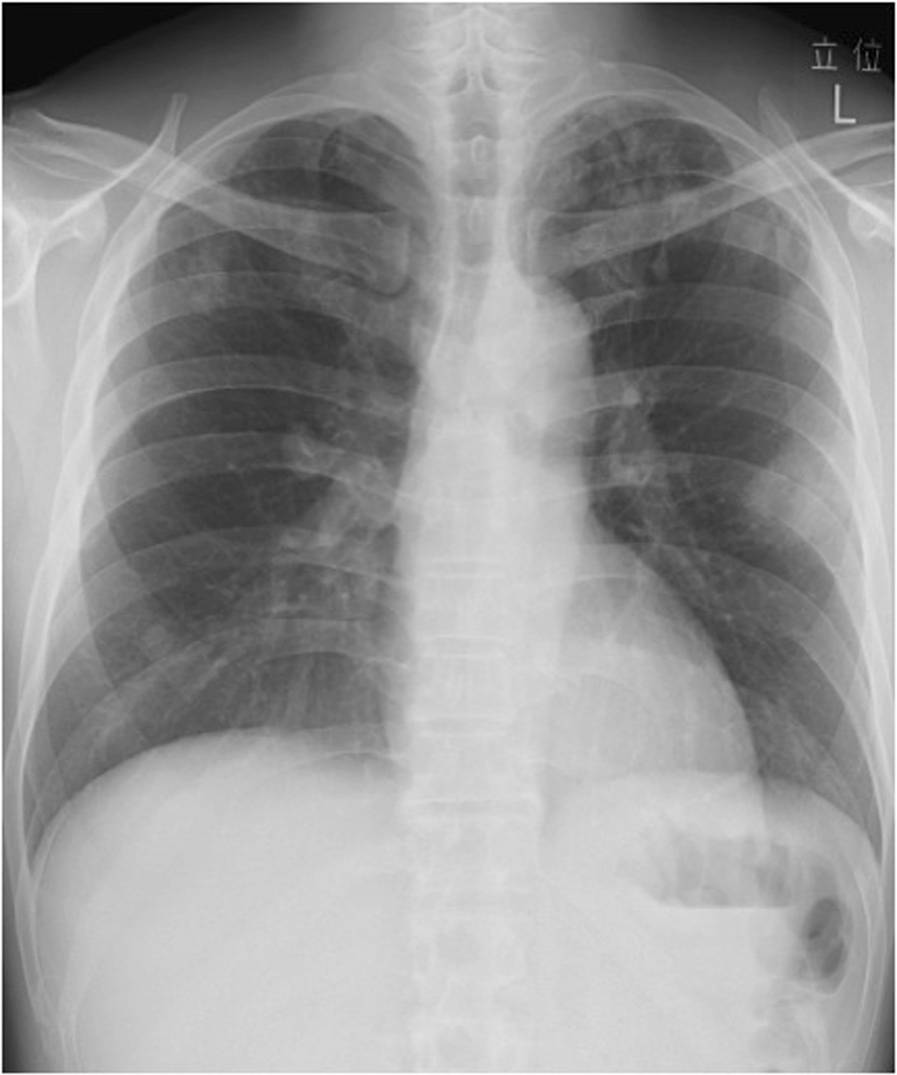
Chest X-ray in June 2006 showed a new solid nodule in the left lung field that gradually enlarged.

**Figure 3:**
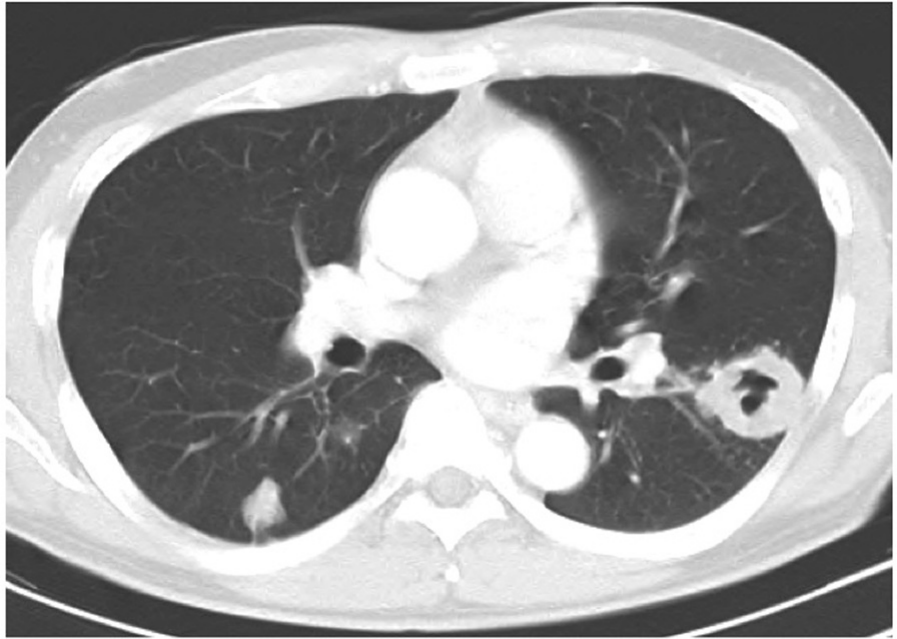
Chest CT in December 2006 showed cavitation of the nodule in the left lung field.

**Figure 4:**
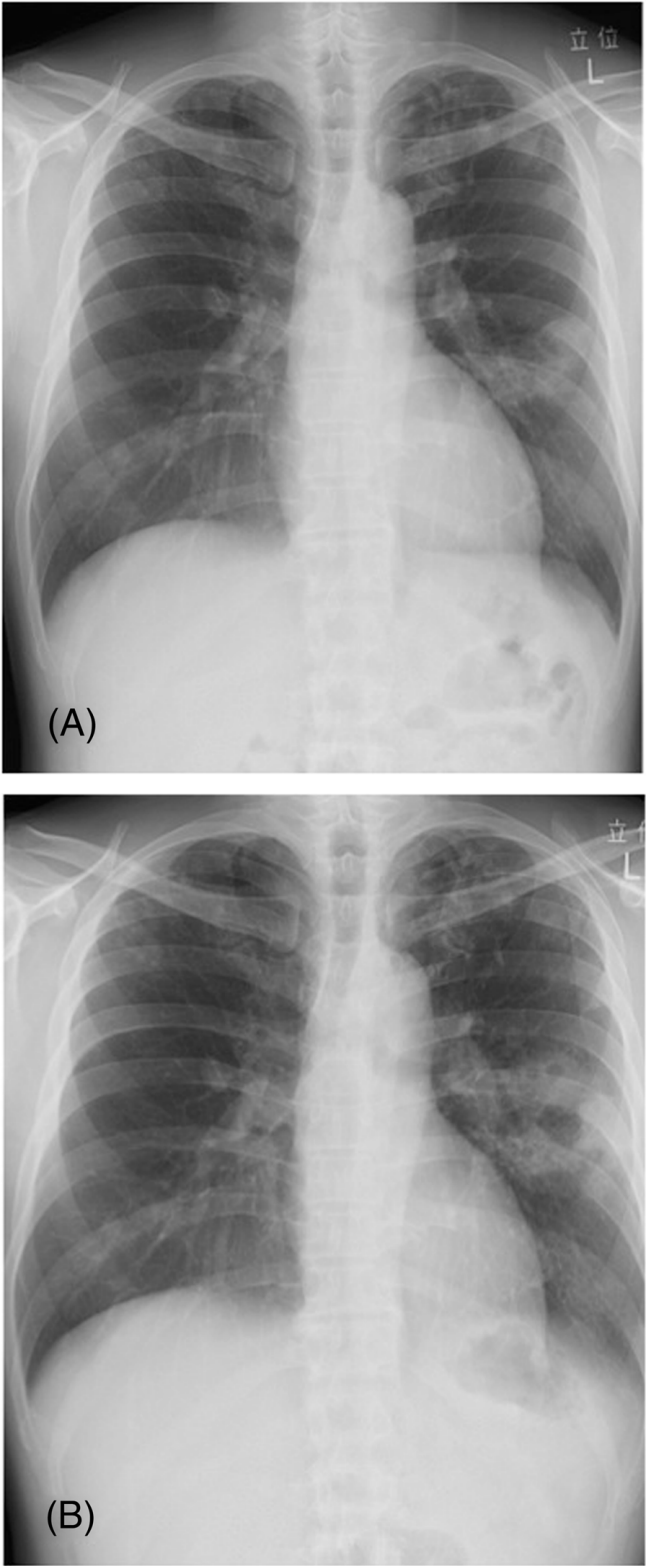
**Chest X-ray in February and May 2007.** Chest X-ray in February 2007 showed that the cavitary lesion in the left lung seemed stable **(A)**. However, in May, the lesion enlarged further **(B)**.

**Figure 5:**
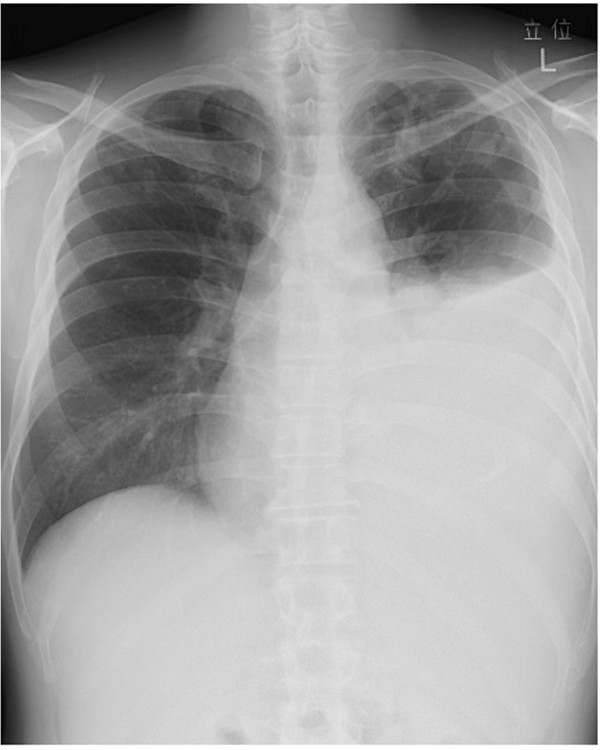
Chest X-ray in June 2007 revealed a left pleural effusion.

**Figure 6:**
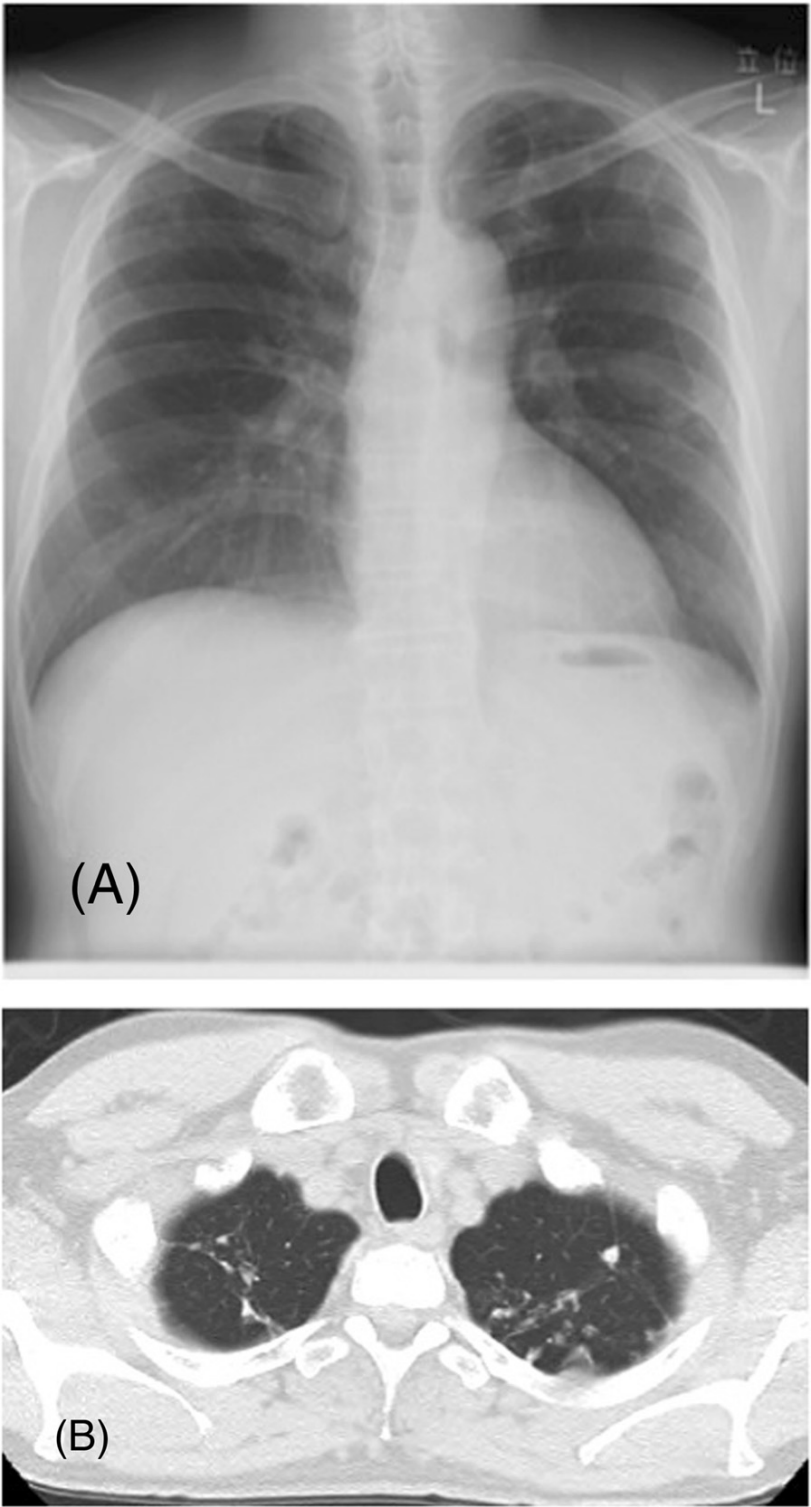
**Chest X-ray and CT after 12 months of treatment.** Chest X-ray after 12 months of treatment **(A)** revealed a decrease in the pleural effusion. Chest CT **(B)** showed that the multifocal nodules tended to disappear.

In June 2006 (7 months later) a new nodule appeared in the left lung field, which gradually enlarged in size (Figure 2). In December 2006 (13 months later), a chest CT revealed cavitation within the nodule (Figure 3). Bronchoscopy was performed once again, but with no positive findings. In February 2007, the cavitary lesion in the left lung seemed stable or slightly reduced in size. In May, the cavitary lesion showed further growth, even though the other nodules did not enlarge in size (Figure 4). The patient was asymptomatic until June 2007, when a small amount of sputum and a fever of 38°C appeared, and chest X-rays revealed a left pleural effusion (Figure 5). Pleural fluid findings showed a predominance of lymphocytes and elevated adenosine deaminase (ADA) levels (57.4 IU/L). Further, PCR-TB of a sputum sample turned out to be positive. Sputum culture in Ogawa’s culture medium for 8 weeks grew four colonies of *Myobacterium tuberculosis*. After treatment with isoniazid (INH), rifampicin (RFP), pyrazinamide (PZA) and ethambutol (EB), all the lesions decreased in size (Figure 6).

## Discussion

Prior to the introduction of chemotherapy for tuberculosis, spontaneous regression of lesions had been regarded as one aspect of a disease’s natural course. Spontaneous regression of lung tuberculosis was reported from the 1950s to 1970s [4-7]. Spontaneous regression of extrapulmonary tuberculosis, in the form of a tuberculous psoas abscess with calcification and intracranial tuberculoma, were also reported in the 1990s [8,9].

In our case, there were two possible mechanisms for the disappearance of the initial nodules prior to the exacerbation of new ones. First is a partial decrease in the immune response in the enlarged lesions. Chronic lung diseases, such as organizing pneumonia (OP) and non-tuberculous *Mycobacterium* infection (NTM), or even an acute infection, such as atypical pneumonia, sometimes present with translocation of the infiltrates or simultaneous occurrence of partial improvement and partial worsening of the infiltration. These phenomena suggest that immune responses may differ in strength, process and phase within a wide lung field. Although our patient presented with nodular shadows, our case may share similar mechanisms of movement of infiltrates as in OP or NTM infection. Second, the local confines of granuloma formation may be broken down only in the lesions, which communicated with an airway, resulting in better oxygen supply for the TB bacilli, and consequent growth of the lesions.

## Conclusion

In conclusion, we report a rare case of pulmonary tuberculosis presenting as multifocal nodules that showed metachronous changes in size, which suggest that the local immune response might be different even in different parts of the same lung field.

## Consent

Written informed consent was obtained from our patient for the publication of this case report and any accompanying images. A copy of the written consent is available for review by the Editor-in-Chief of this journal.

## Competing interests

The authors declare that they have no competing interests.

## Authors’ contributions

TI, ST, KW, AM and YH participated in the clinical diagnosis. MK supervised the concept and design of the manuscript and gave final approval of the version to be published. All authors read and approved the final manuscript.
